# Replication Kinetics of a Candidate Live-Attenuated Vaccine for Cache Valley Virus in *Aedes albopictus*

**DOI:** 10.1089/vbz.2022.0053

**Published:** 2022-11-09

**Authors:** Victoria B. Ayers, Yan-Jang S. Huang, James I. Dunlop, Alain Kohl, Benjamin Brennan, Stephen Higgs, Dana L. Vanlandingham

**Affiliations:** ^1^ Department of Diagnostic Medicine/Pathobiology, College of Veterinary Medicine, Kansas State University, Manhattan, Kansas, USA.; ^2^ Biosecurity Research Institute, Kansas State University, Manhattan, Kansas, USA.; ^3^ MRC-University of Glasgow Centre for Virus Research, Glasgow, United Kingdom.

**Keywords:** Cache Valley virus, live-attenuated vaccine, *Aedes albopictus*

## Abstract

**Background:**

The emergence or re-emergence of several orthobunyaviruses (order: *Bunyavirales*; family: *Peribunyaviridae*), including Cache Valley virus (CVV) and Oropouche virus, warrants the development and evaluation of candidate live-attenuated vaccines (LAVs). Ideally, these vaccines would elicit long-lasting immunity with one single immunization.

**Materials and Methods:**

Since the deletion of two virulence factors, NSs and NSm, has been shown to attenuate the virulence phenotype of orthobunyaviruses, phleboviruses, and nairoviruses, genetic manipulation of the viral genome is considered an effective strategy for the rational design of candidate LAVs for bunyaviruses across multiple families. In addition, the deletion of Rift Valley fever virus NSs and NSm genes has been shown to reduce transmission by mosquitoes.

**Results:**

In this study, the ability of a CVV mutant lacking the NSs and NSm genes (2delCVV) to replicate in intrathoracically injected *Aedes albopictus* was compared with the parental wild-type CVV (wtCVV) 6V633 strain. In contrast to the robust replication of wtCVV in injected mosquitoes, the multiplication kinetics of the 2delCVV mutant was reduced by more than a 100-fold.

**Conclusion:**

These results suggest that the deletion of NSm and NSs genes is a feasible approach to rationally design candidate orthobunyavirus LAVs that are highly attenuated in mosquitoes and, therefore, pose little risk of reversion to virulence and transmission.

## Introduction

The *Orthobunyavirus* genus contains several human and animal pathogens. One emerging virus in this genera is Cache Valley virus (CVV), which is a zoonotic orthobunyavirus that causes abortions and teratogenic effects in large animals and rare but severe encephalitis in humans (Campbell et al, 2006; Nguyen et al, 2013; Sexton et al, 1997; Wilson et al, 2017; Yang et al, 2018). CVV has been isolated from several mosquito species and animals such as *Aedes*, *Anopheles*, *Coquillettidia*, *Culiseta*, *Ochlerotatus*, and *Psorophora* (Andreadis et al, 2014; Yang et al, 2018) and cattle, sheep, and caribou (Calisher et al 1986). Serological evidence of potential infections has also been found in horse, elk, and white-tailed deer (Calisher et al, 1986).

Although CVV has caused significant agroeconomic losses, no research has truly investigated the economic burden from the disease (Lopez et al, 2021). Currently, there are no licensed vaccines commercially available for the control of CVV in animals or humans. As observed with several viral diseases, live-attenuated vaccines (LAVs) are the most effective tool for disease control (Davis et al, 2019). This is because many LAVs can potentially elicit a long-lasting protective immunity with one single immunization such as the yellow fever (YF) 17D LAV (Collins and Barrett, 2017).

A live-attenuated candidate vaccine for CVV was evaluated in this study to not only aid in the event of an outbreak but also to advance our knowledge in vaccine development for other related bunyaviruses. Because all orthobunyaviruses are evolutionarily related and possess common motifs encoded by shared sequences, the attenuation process of CVV caused by genetic deletions can be a proof of concept for the development of candidate LAV for other related viruses such as La Crosse virus. The gene deletions are hypothesized to attenuate the virulence phenotype of CVV, thus restricting the vaccine viruses' ability to replicate in mosquitoes.

Most live-attenuated arbovirus vaccines with excellent safety profiles have been shown to lose the ability to infect and replicate in mosquitoes to prevent the possible transmission due to the deployment of LAVs in the field (Monath et al, 2020). For example, two highly effective LAVs for arboviruses, YF 17D vaccine and Japanese encephalitis SA14-14-2 vaccine, are unable to replicate and disseminate in mosquitoes (Chen and Beaty, 1982; Danet et al, 2019). In contrast, vaccine strains that did not reach a safe level of attenuation can often infect, multiply, and disseminate in mosquitoes.

One example of this was the Venezuelan equine encephalitis virus LAV TC-83, which is capable of infecting biting mosquitoes after equine vaccination (Pedersen et al, 1972). Similarly, the partially attenuated Rift Valley fever virus (RVFV) Smithburn LAV was transmitted by *Culex pipiens* and caused outbreaks in Egypt, which demonstrates the risk for vaccine viruses to mutate and ultimately revert to the virulence phenotype (Ahmed Kamal, 2011; Kamal et al, 2018).

The attenuated phenotype of LAVs for flaviviruses and alphaviruses in mosquitoes has been investigated in multiple systems (Davis et al, 2022; Gorchakov et al, 2012; Kaiser et al, 2019a; Kaiser et al, 2019b; McElroy et al, 2008; McElroy et al, 2006; McElroy et al, 2005; Plante et al, 2015; Rossi et al, 2020; Wang et al, 2017), whereas there is a major gap in knowledge of the attenuation process of bunyaviruses in mosquitoes. Specific genetic loci that govern the infection process of bunyaviruses across multiple genera are not well understood, warranting the investigation of the attenuated phenotype of CVV caused by the simultaneous deletion of the NSs and NSm genes.

To date, the virulence phenotype of multiple orthobunyaviruses, including Bunyamwera virus (BUNV) and Schmallenberg virus (SBV), has been attenuated by the deliberate removal of virulence factors in the viral genome (Kraatz et al, 2015; Szemiel et al, 2012). The orthobunyavirus genome consists of three negative-sense RNA segments, small (S), medium (M), and large (L), which code for various structural and nonstructural (NS) proteins (Hughes et al, 2020). Orthobunyaviruses have two known virulence factors, the NSs gene encoded in the S segment and the NSm gene encoded in the M segment (Elliott, 2014).

Deletion of either virulence factor was previously sufficient for virulence attenuation, as demonstrated with BUNV, the prototype orthobunyavirus (Bridgen et al, 2001; Szemiel et al, 2012). The simultaneous deletion of NSs and NSm genes fully attenuated the virulence phenotype of SBV, an emerging orthobunyavirus, in immunocompromised mice (Kraatz et al, 2015). In addition, in a previous study, we demonstrated that sheep inoculated with the candidate vaccine 2delCVV, lacking the NSs and NSm genes, developed a robust neutralizing antibody response with titers that would likely protect them from infection with wild-type CVV (wtCVV) (Ayers et al, 2022).

Previously, Seligman and Gould (2004) raised concerns regarding the potential for arbovirus LAVs to infect mosquitoes. Herein, we determine the capacity of a candidate CVV LAV strain that lacks the virulence factor NSs and NSm genes (2delCVV) to replicate in mosquitoes as compared with the CVV 6V633 wild-type strain. In a previous study, CVV did not require NSs to support replication in mosquito cell lines Aag2, similar to the deletion of NSm in Oropouche virus; therefore, attenuation of CVV in mosquitoes may be possible through the simultaneous deletion of NSs and NSm (Dunlop et al, 2018; Tilston-Lunel et al, 2015). The deletion of the virulence factors has also reduced the ability of RVFV to enter, replicate, and disseminate from the midgut epithelial cells (Kading et al, 2014). In addition, the attenuated phenotype of BUNV NSs deletion mutant has also included the reduced multiplication kinetics in infected mosquitoes (Szemiel et al, 2012).

We have previously shown that *Aedes* (*Ae.*) *albopictus* and *Culex* (*Cx.*) *tarsalis* are competent vectors for CVV (Ayers et al, 2019; Ayers et al, 2018). *Ae. albopictus* has a broader geographic distribution throughout North America and has recently been found to be infected with CVV in New York State, potentially indicating a northeastern U.S. expansion in the range of this virus (Dieme et al, 2022; Kamal et al, 2018).

Therefore, *Ae. albopictus* mosquitoes are considered an important vector for the endemic transmission of CVV and an appropriate model system to study the attenuating effect caused by the simultaneous deletion of NSs and NSm. Because the multiplication kinetics of 2delCVV is significantly reduced and does not reach the threshold infectivity required for mosquito *per os* infection (data not shown), *Ae. albopictus* were intrathoracically inoculated with either CVV 6V633 or 2delCVV to investigate the replication kinetics by bypassing the viral entry process. Our results demonstrated that the double deletion mutant of CVV displayed an attenuated phenotype based on the lower multiplication kinetics when compared with the wild-type 6V633 CVV strain.

## Materials and Methods

### Cells and viruses

The prototype 6V633 strain of CVV, originally isolated from infected *Culiseta inornata* in Cache Valley, Utah, in 1956 (Holden and Hess, 1959), was used as the wild-type virus for the intrathoracic inoculation. The strain was obtained from the laboratory collection of Dr. Richard M. Elliot (Watret et al, 1985). In a previously published study, the sequences of all three genomic segments were determined (GenBank acc. nos. KX100133.1, KX100134.1, and KX100135.1) (Groseth et al, 2017). Virus stocks were propagated and titered in African green monkey kidney epithelial Vero 76 cells that were maintained in Leibovitz's L-15 media (Thermo Fisher Scientific, Waltham, MA) supplemented with 10% fetal bovine serum, 10% tryptose phosphate broth, penicillin/streptomycin, and l-glutamine (Ayers et al, 2019; Ayers et al, 2018; Huang et al, 2015). The 2delCVV candidate vaccine was created using the same methods as previously described (Ayers et al, 2022; Dunlop et al, 2018).

### Mosquitoes and challenge

Intrathoracic injection of mosquitoes was performed with 7- to 10-day-old *Ae. albopictus* (F_4_), which were derived from eggs collected from the city of Trenton, Mercer County, NJ, in July 2016. Mosquitoes were reared at 28°C, relative humidity of 80%, and a 12 h light:12 h dark photoperiod.

For intrathoracic inoculation, mosquitoes were cold anesthetized and inoculated with ∼0.5 μL of either the CVV 6V633 strain or the 2delCVV strain, as previously described (Huang et al, 2020). The experimentally challenged mosquitoes were maintained at conditions as described earlier for 7 days. Up to three mosquitoes were collected after injection and titrated to confirm the presence of infectious viruses. Intrathoracically inoculated mosquitoes were then collected by mechanical aspiration at 7 days postinfection (dpi) for analysis.

### Detection of infectious viruses

Infection status of each mosquito was determined by the detection of CVV using the tissue culture infectious dose 50% (TCID_50_)-based titration method with Vero76 cells, as previously described (Ayers et al, 2019; Higgs et al, 2006; Huang et al, 2015). Infectivity of mosquitoes was used to determine the infection rate and multiplication kinetics of the wtCVV 6V633 strain and the 2delCVV strain. Infection rates were then calculated using the percentage of infected mosquitoes among all mosquitoes tested at each time point. Growth kinetics of CVV in infected mosquitoes was determined based on the titers of CVV in the whole mosquitoes at 7 dpi.

### Statistical analysis

The infection rate percentages were compared using chi-squared test with Yate's correction. Titers of infected mosquitoes were compared with Mann–Whitney rank sum test between the two groups with the infectious titers after non-normal distribution. All statistical analyses were conducted using GraphPad (San Diego, CA) and Excel software (Redmond, WA).

## Results

**FIG. 1.**
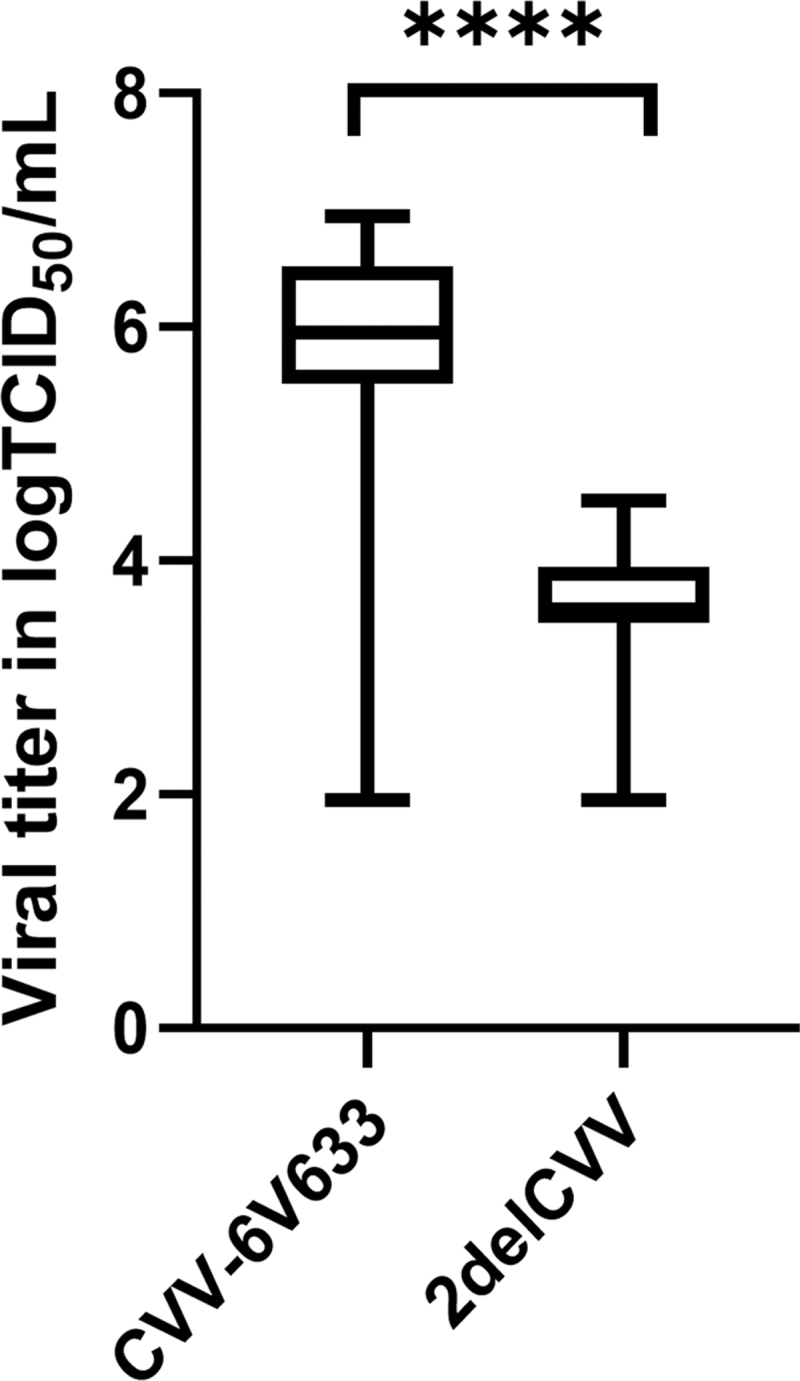
Titers of CVV in *Aedes albopictus* at 7 dpi. A Mann–Whitney test was used to compare the viral titers between the two groups of mosquitoes (****p < 0.001). The maximum and minimum values are displayed by the *vertical lines* connecting the largest and smallest viral titer in the data set. The *horizontal bar* represents the median titer of whole mosquitoes. CVV, Cache Valley virus; dpi, days postinfection.

As expected, intrathoracic injection with either the CVV 6V633 strain or the 2delCVV candidate vaccine led to the establishment of infection in *Ae. albopictus*. There was no significant difference in the infection rates produced by 2delCVV and the CVV 6V633 strain at 7 dpi (CVV 6V633: 83.1% [49/59] vs. 2delCVV: 97.5% [39/40], chi-squared test with Yates's correction: *χ*^2^ = 3.682, *df* = 1, *p* = 0.0550) (Table 1). Mosquitoes injected with the 2delCVV candidate vaccine had significantly lower infectivity at 7 dpi than mosquitoes infected with wtCVV (CVV 6V633: 6.0 log_10_TCID_50_/mL vs. 2delCVV [median titer]: 3.5 log_10_TCID_50_/mL; Mann–Whitney test: *U* = 75.5, *p* < 0.0010) (Fig. 1). In conclusion, the deletion of NSs and NSm genes significantly reduces the multiplication kinetics of CVV in mosquitoes.

**Table 1.** Comparison of Infection Rates in *Aedes albopictus* Mosquitoes Intrathoracically Injected with CVV 6V633 or 2delCVV

Group	Mosquitoes tested	7 dpi
CVV-6V633	59	83.1% (49/59)
2delCVV	40	97.5% (39/40)

CVV, Cache Valley virus; dpi, days postinfection.

## Discussion

The development of safe and immunogenic LAVs for emerging bunyaviruses with minimal potential to revert to the virulence phenotype has become a priority for human and animal health in multiple geographic regions. It is also important that the bunyavirus LAVs exhibit reduced infection, dissemination, and multiplication kinetics in mosquitoes, preventing mosquitoes from transmitting vaccine viruses to livestock or humans. Data generated in this study demonstrated that the 2delCVV vaccine candidate was restricted in its replication in intrathoracically inoculated *Ae. albopictus* compared with the CVV 6V633 wild-type strain.

Along with the reduced multiplication kinetics of the 2delCVV strain in the vertebrate host SFT-R cells (Dunlop et al, 2018), this candidate CVV LAV has a lower potential of transmission by mosquitoes, similar to the attenuated phenotype of the YF 17D vaccine strain in *Ae. aegypti*. It is important to note that the simultaneous deletion of NSs and NSm genes has been proven to significantly impair the multiplication kinetics of CVV and related orthobunyaviruses in vertebrate hosts (Bridgen et al, 2001; Kraatz et al, 2015). The likelihood of 2delCVV to multiply to high viremic titers is low.

The low multiplication kinetics of 2delCVV in mosquitoes observed in this study further suggests the utility of the 2delCVV mutant as a candidate vaccine. In the unlikely but possible event that a mosquito acquires viremic bloodmeal containing the 2delCVV in nature, the low multiplication kinetics will limit the dissemination and minimize the likelihood of transmission. In addition, the yield of progeny virions is low and consequently can further reduce the risk of reassortment between the 2delCVV and wtCVV.

Although the YF 17D vaccine was able to infect mosquitoes, it was unable to disseminate to the secondary tissues and failed to transmit to a novel host (Danet et al, 2019). This suggests that the midgut escape barrier and the midgut infection barrier restrict this LAV from replication and transmission. Similarly, *Ae. aegypti* and *Ae. albopictus* were orally challenged with ChimeriVax vaccine candidates and resulted in such low titers that it would be unlikely for these mosquitoes to facilitate transmission (Higgs et al, 2006). Restriction of 2delCVV was observed in this study.

But the detailed mechanism(s) of attenuation remains undefined. The most plausible hypothesis is that the simultaneous deletion of NSs and NSm interferes with the virus–vector interactions required for multiplication of CVV after the establishment of infection in mosquitoes. Furthermore, it is unlikely that the 2delCVV mutant is defective in the cell entry process because the 2delCVV mutant has the same coding sequence for the two structural proteins and showed no demonstrable difference in the infection rate compared with the parental wild-type 6V633 strain.

The findings in this study also suggest the NSs and NSm genes are necessary for efficient growth in *Aedes* species mosquitoes. The specific role of NSs and NSm in mosquitoes still needs to be defined since NSs is not essential for viral growth in cell culture and NSm has been hypothesized to be dispensable for virus replication in mosquito cell lines (Elliott, 2014; Tilston-Lunel et al, 2015). Although unnecessary for viral growth in cell lines, the presence of these genes suggest they are necessary to overcome the cellular defenses in the midgut. In a previous study, the deletion of NSs in BUNV was unable to bypass the cellular defenses; however, when these barriers were overcome the vaccine viruses were capable of spreading to the secondary tissues and salivary glands (Szemiel et al, 2012).

More recently, a human vaccine candidate for RVFV lacking the NSm gene was unable to infect, replicate, or be transmitted by multiple mosquito species (Campbell et al, 2022). Therefore, the NSs and NSm genes appear to be required for the efficient replication of bunyaviruses in mosquitoes. In conclusion, 2delCVV exhibited an attenuated phenotype in mosquitoes through the reduced replication kinetics observed when compared with the wild-type strain and this attenuated phenotype reflects the functions of the NSs and NSm genes.
